# Reoperating on the Y-incision root enlargement: Deconstruction and reconstruction

**DOI:** 10.1016/j.xjtc.2024.01.008

**Published:** 2024-01-19

**Authors:** Stephen M. Spindel, Reginald E. Du, Katrina J. Jiang, Jasmine Su

**Affiliations:** a Section of Cardiothoracic Surgery, Department of Surgery, Ochsner Medical Center, New Orleans, La; b The University of Queensland Medical School, Ochsner Clinical School, New Orleans, La; c The University of Massachusetts, Amherst, Mass

**Figure:**
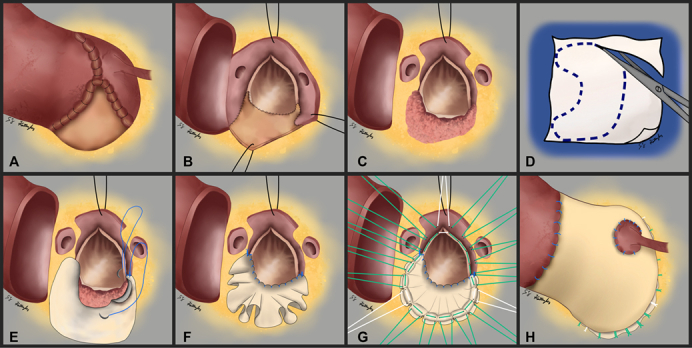
Y-incision root resection with reconstruction via oversized patch for aortomitral curtain.

**Central Message**

Takedown of a prior Y-incision root enlargement leaves behind a small LVOT and a root requiring an oversized-patch reconstruction of the aortomitral curtain, as demonstrated in Video 1.

The Y-incision technique is a recent addition to the aortic root enlargement repertoire that is gaining fame, partly due to its ability to increase the annulus by 3 or 4 valve sizes.^1^, ^2^, ^3^ The operation utilizes a *Y*-shaped incision into the aortomitral curtain followed by placement of a rectangular patch for aortic annular enlargement.^1^^,^^2^ By sparing the anterior mitral leaflet, the left ventricular outflow tract (LVOT) remains unchanged and the risk of mitral regurgitation is reduced.^3^ The application of this technique is becoming popular, yet no literature discusses the challenges faced with reoperating on a Y-incision root enlargement.

Herein, we discuss a case where an 18-mm aortic annulus was upsized to 23 mm using the Y-incision technique, yet 6 months postoperatively an aortic root abscess developed. Institutional review board approval was not required and informed written consent was obtained to include patient information in Video 1. Resection of the Y-incision patch generates the dilemma of having the original small LVOT and a now-ravaged aortomitral curtain (Figures 1 and 2). Additionally, patch removal leaves behind a large aortic root defect that extends inferior to the left coronary ostium. Aortic root replacement and aortomitral curtain reconstruction are required, yet a small valved-conduit replacement is undesirable. To achieve a larger valved-conduit root replacement in this situation, focus is placed on the aortomitral curtain reconstruction (Video 1).

**Figure 1:**
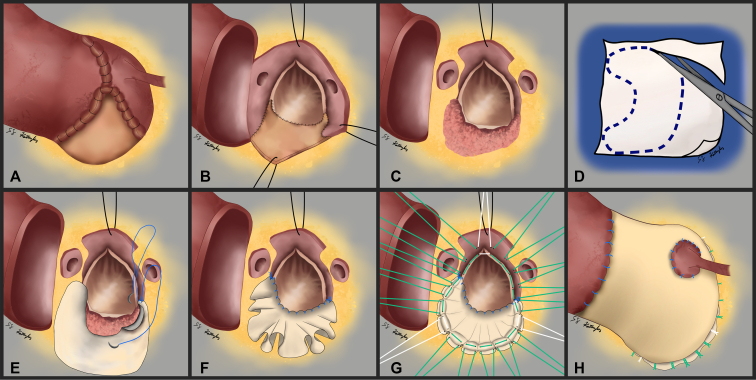
Stepwise deconstruction of a prior Y-incision aortic root enlargement and subsequent aortomitral curtain and aortic root reconstruction. A, Aortotomy performed at the superior edge of the root enlargement patch. B, The aortic prosthesis is removed, showing the patch attachments. C, Resection of the patch leaves minimal aortomitral curtain remaining. D, An arc or crescent shaped patch is created from bovine pericardium. E, The patch-to-LVOT anastomosis is initiated at the right fibrous trigone using a continuous suture. F, Completed patch anastomosis highlights the redundancy needed for aortomitral curtain reconstruction in a small LVOT. G, Aortic annular sutures are placed, creating the neoaortic annulus on the patch. H, Completion of the root replacement displays the outwardly expanded aortic root secondary to an enlarged aortomitral curtain.

**Figure 2:**
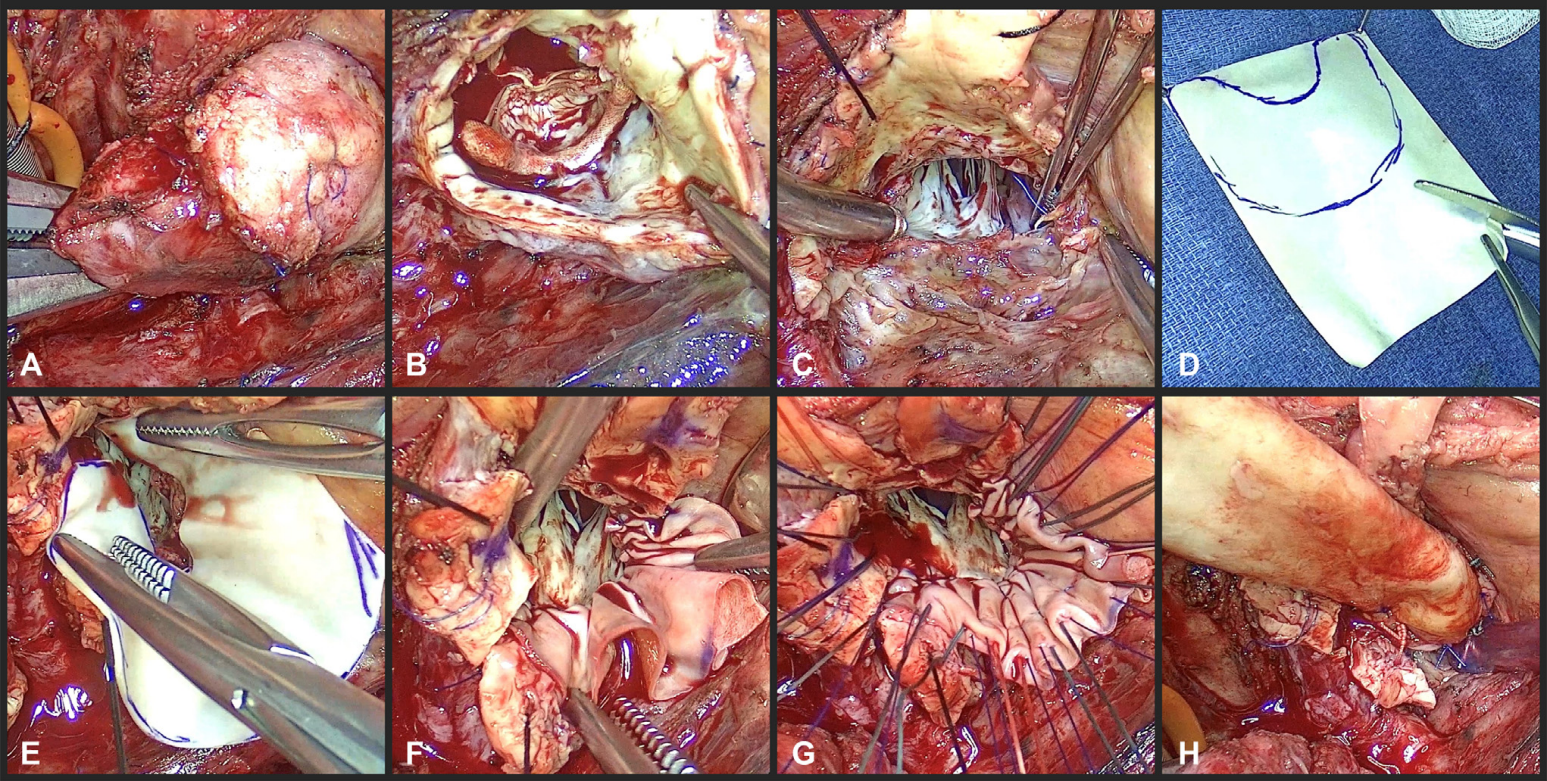
Intraoperative images of the stepwise deconstruction of a prior Y-incision aortic root enlargement and subsequent reconstruction. A, Aortotomy performed at the superior edge of the root enlargement patch. B, The aortic prosthesis is explanted followed by patch removal. C, Resection of the patch leaves minimal aortomitral curtain remaining. D, An arc or crescent shaped patch is created from bovine pericardium. E, The inner diameter of the patch matches the LVOT, while the outer diameter maintains an enlarged aortic annulus. F, Completed patch anastomosis highlights the redundancy needed for aortomitral curtain reconstruction in a small LVOT. G, Aortic annular sutures are placed, creating the neoaortic annulus on the patch. H, Completion of the root replacement displays the outwardly expanded aortic root secondary to an enlarged aortomitral curtain.

The neoaortic annulus, and thus valved-conduit size, is dictated by the patch dimensions of the rebuilt aortomitral curtain. An oversized patch is critical and shaped as an arc or crescent, having a smaller inner curve and a larger outer curve. Specifically, for a patient with prior Y-incision root enlargement who has a small LVOT and no residual aortomitral curtain, the patch is tailored to have a small inner diameter and an excessively larger outer diameter, allowing for redundancy. Although the inner diameter matches the native LVOT, the outer diameter controls the neoaortic annular circumference. After the inner curve is anastomosed to the fibrous trigones and mitral annulus, the aortic annular sutures are placed along the redundant outer curve. This reconstructs the aortomitral curtain while maintaining the previous root enlargement. The remaining root replacement steps are performed in standard fashion. Here, a 24-mm homograft was implanted with an uneventful postoperative course and satisfactory echocardiogram (Figure E1).

## Conflict of Interest Statement

The authors reported no conflicts of interest.

The *Journal* policy requires editors and reviewers to disclose conflicts of interest and to decline handling manuscripts for which they may have a conflict of interest. The editors and reviewers of this article have no conflicts of interest.

## Figures and Tables

**Figure E1:**
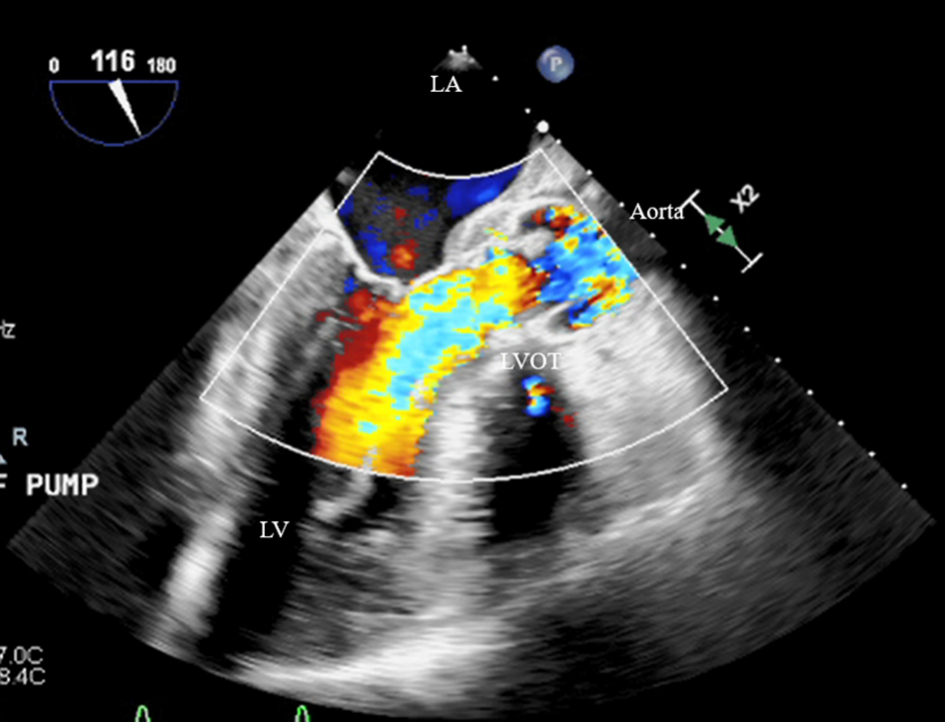
Postbypass transesophageal echocardiogram showing an aortic transvalvular mean gradient of 10 mm Hg, no aortic regurgitation, no left ventricular outflow tract (*LVOT*) obstruction, and mild mitral regurgitation. *LA*, Left atrium; *LV*, left ventricle.

## Supplementary Data

Video 1 Intraoperative video illustrating the deconstruction of a prior Y-incision aortic root enlargement and its reconstruction with an oversized patch for the aortomitral curtain. Video available at: https://www.jtcvs.org/article/S2666-2507(24)00010-5/fulltext.
