# Supplementary figures and images for: Reoperating on the Y-incision root enlargement: Deconstruction and reconstruction
Source: JTCVS Tech. 2024 Jan 19;24:23–6. doi: 10.1016/j.xjtc.2024.01.008 (PMC11145066; doi:10.1016/j.xjtc.2024.01.008)

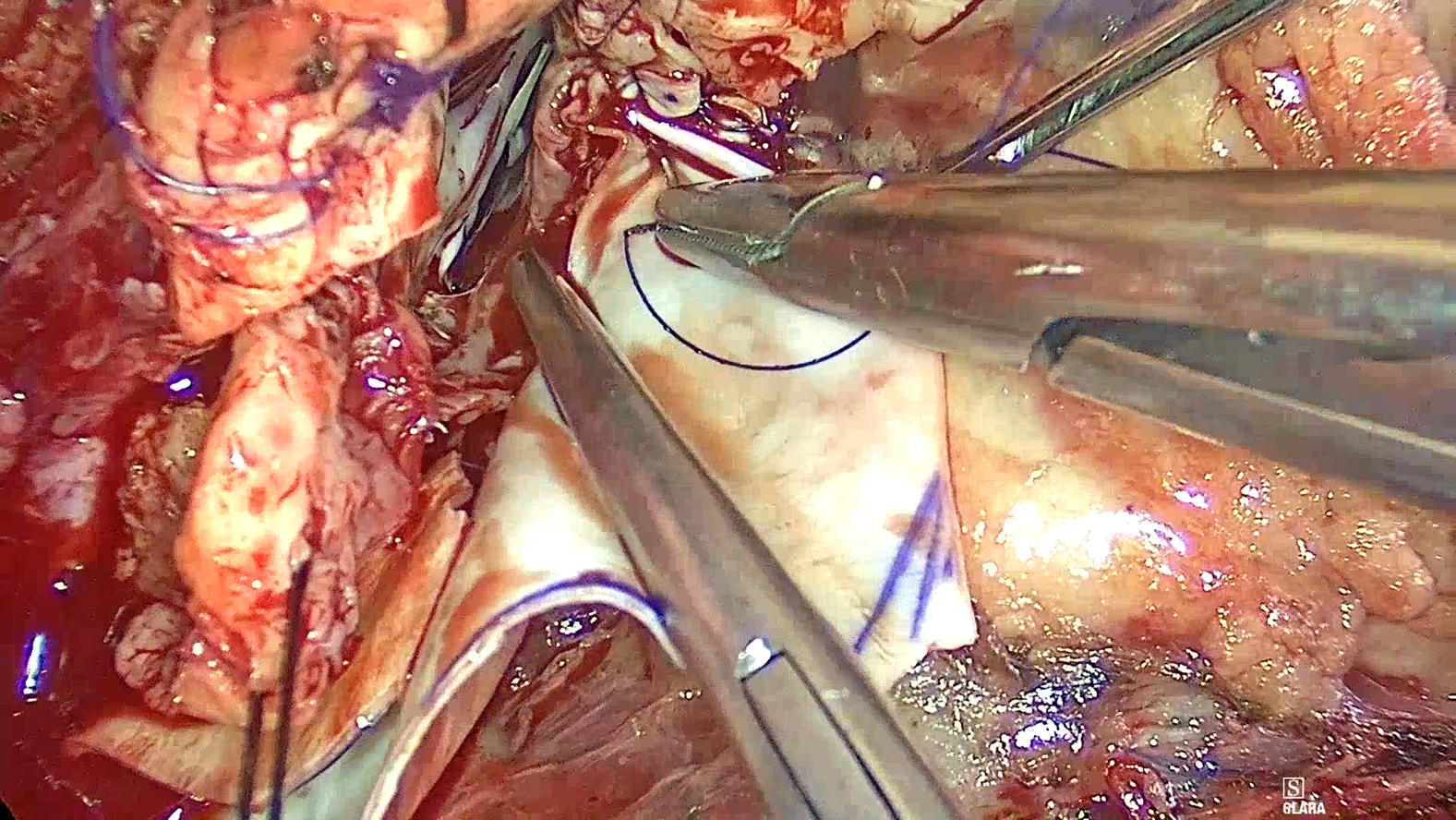

Supplement: Video 1 — Intraoperative video illustrating the deconstruction of a prior Y-incision aortic root enlargement and its reconstruction with an oversized patch for the aortomitral curtain. Video available at: https://www.jtcvs.org/article/S2666-2507(24)00010-5/fulltext. [file fx2.jpg]
